# Hospital Co-Operation

**Published:** 1923-10

**Authors:** C. M. Power

**Affiliations:** Secretary of Westminster Hospital


### HOSPITAL CO-OPERATION.

### WHAT WESTMINSTER IS DOING.

By C. M. POWER, M.C., Secretary of Westminster Hospital.

I ARRIVED at Westminster Hospital to find that. in common with all other hospitals, the greatest needs were extensions, alterations and improvements, and—money. The need for extensions compelled the hospital to close temporarily on July 1, and arrangements have been made for the following work to be carried out:—A new nurses' home in Queen Anne's Gate (a few hundred yards from the hospital) to accommodate all sisters and nurses who have hitherto lived in cramped and unhealthy quarters in the hospital; new maternity ward, wards for ear, nose, and throat cases; a second operating theatre for in-patients; an operating theatre for minor operations in the out-patients' department; new and enlarged departments for electrical X-ray and massage treatment; extension of the pathological department and the department for treatment of venereal diseases; the installation of an electro cardio graphic apparatus; new kitchens; improved accommodation for the resident medical staff; and many general improvements, such as the provision of central heating, and thorough repair and re-decoration of the hospital building inside and out. A fourth storey is to be erected to provide private rooms for sick nurses and the reception of paying patients to meet the constant demand for such accommodation.

### The Results of Propaganda.

Although in a unique position adjacent to the Houses of Parliament and Westminster Abbey, with tens of thousands of people passing daily, the hospital appears to be little known. Therefore to bring to the notice of the general public the work and needs of the hospital various steps have been taken. In conjunction with St. George's Hospital a regular and systematic house-to-house canvass is being made in the areas inhabited by those using the two hospitals, and a special appeal department has been set up at the hospital to seek the regular support of citizens and business men of Westminster. This is done not only by letter but by personal visits from representatives of the hospital. The result is that the endowments of the hospital have increased during the past two years by £14,600, and the ordinary annual income has increased by £7,500. Towards the extensions and alterations which are to cost approximately £45,000, £18,000 has been received or promised.

### The Westminster Co-operative Committee.

I am keenly interested in any movement which will lead to co-operation in work and in appeals for support of neighbouring hospitals. We have in Westminster a committee representative of the City's business men, residents, Government officers, religious and charitable bodies over which the Mayor presides. The efforts of this committee, of which I am secretary, are directed to co-operating the work and appeals of all hospitals in Westminster. It was most useful and successful during the Combined Hospitals' Appeal last year, when by careful propaganda considerably over £15,000 was raised in Westminster alone The value of such co-operation is reflected in a small way in the success of the Flag Day on May 24, 1922, when as the result of the nine hospitals of the city working in complete accord and their efforts being co-ordinated and administered from Westminster Hospital, £4,238 was collected. This constituted the largest amount collected by any group of hospitals in London. It takes time, but I hope to see within the course of a few years all the hospitals in the City of Westminster working together in their treatment of the sick poor and appealing together as one body for the support of their citizens. Also, I hope to see, in time, the co-operation in treating the sick of the cottage hospitals, the county hospitals and the great institutions in the towns with their medical schools.

### The R.A.M.C. System.

Undoubtedly the system which would be of the greatest value to the public is that of the Royal Army Medical Service, which worked so splendidly during the War, whereby minor ailments and injuries were dealt with at the Field Ambulance Stations, more severe cases at the Casualty Clearing Stations, and obscure and difficult ones at the Consulting Hospitals. At present many beds in our great London hospitals are occupied by patients from the country suffering from ailments which could readily be treated in the cottage hospital. On the other hand, there are long waiting lists of patients suffering from obscure or serious maladies for whom the use of elaborate apparatus and bacteriological departments are necessary.

---

### THE GERM OF MEASLES.

One of the most paradoxical facts in medicine is our total lack of knowledge of the bacterial causes of some of the commonest diseases, whereas in many of the rarest we know every detail of their causal germ. Therefore it seems almost too good to be true that some one has at last discovered the cause of measles and possibly of scarlet fever also. In a recent number of the *Journal of Tropical Medicine and Hygiene*, Dr. David Thomson describes a coccus which he has found almost constantly in the air passages of these cases, with which he has at least produced very active catarrhal symptoms when transferring it to laboratory animals. This does not, of course, prove that the coccus causes measles and Dr. Thomson does not indeed claim as much, for the only way to clinch the matter is to produce the complete disease at least in animals by direct use of the germs. Also, when reading of germ discoveries, the public should be made clearly to understand that because the germ is identified the fact does not necessarily mean that treatment is simplified or the incidence of the disease reduced. Nevertheless, we may earnestly hope that the preliminary announcement already made is the forerunner both of a complete proof and an ample protective scheme.

